# Rapid and Highly Sensitive Detection of *Leishmania* by Combining Recombinase Polymerase Amplification and Solution-Processed Oxide Thin-Film Transistor Technology

**DOI:** 10.3390/bios13080765

**Published:** 2023-07-28

**Authors:** Weidong Wu, Manish Biyani, Daisuke Hirose, Yuzuru Takamura

**Affiliations:** 1 School of Materials Science, Japan Advanced Institute of Science and Technology, Nomi 923-1292, Ishikawa, Japan; weidong.wu@ml.jaist.ac.jp (W.W.); biyani@jaist.ac.jp (M.B.); d-hirose@jaist.ac.jp (D.H.); 2 BioSeeds Corporation, JAIST Venture, Ishikawa Create Laboratory 202, Asahidai 2-13, Nomi 923-1292, Ishikawa, Japan

**Keywords:** oxide thin-film transistor biosensors, recombinase polymerase amplification, *Leishmania HSP70*, point-of-care diagnostics, DNA rapid detection

## Abstract

Nucleic acid detection is widely used to identify infectious diseases and ensure food safety. However, conventional PCR-based techniques are time consuming. Thus, this study aims to combine recombinase polymerase amplification (RPA), which enables the rapid amplification of even trace amounts of nucleic acid fragments within 10–40 min at 37–42 °C, and solution-processed oxide thin-film transistor (TFT) technology, which exhibits high detection sensitivity, to detect *Leishmania*. A single-stranded anti-probe was incorporated into the RPA primer to facilitate effective hybridization between the RPA product and the immobilized probe on the solution-processed oxide TFT. The RPA-amplified product carrying an anti-probe enabled specific binding to the chip surface. Changes in current were monitored before and after sample incubation to identify the target nucleic acids in the samples accurately. The proposed method achieved a remarkable limit of detection of 10^1^ copies/μL of the *Leishmania HSP70* fragment within 30 min. The design of the probes on the solution-processed oxide TFT surface and the anti-probe simplified the detection of other target nucleic acids, eliminating the need to denature DNA double-strands for specific binding during nucleic acid detection. Thus, the novel method offers the advantage of requiring minimal reagent resources and eliminates the need for complex procedures.

## 1. Introduction

Nucleic acid detection has become increasingly crucial for the diagnosis of infectious diseases [1,2,3]. It has a higher detection sensitivity and broader applicability than traditional antigen and antibody detection methods. For example, the use of Smart Probes (SP), which are attached to a fluorophore dye, for the specific recognition of nucleic acid markers such as miRNA levels in cancer cells has been reported [4,5,6]. Over the past three years, SARS-CoV-2 has spread worldwide, and nucleic acid detection has played an important role in epidemic detection and prevention [2,7]. However, conventional PCR-based nucleic acid detection methods have some shortcomings, such as requiring laboratory settings, professional operation, and a long detection time of over 2 h [8]. Therefore, innovative nucleic acid detection technologies that are highly sensitive, affordable, and easy to use need to be developed. Such features are instrumental in enabling rapid and efficient disease diagnosis in environments constrained by limited resources.

Over the past decade, Si-based label-free and graphene field-effect transistors (GFETs) have emerged as fundamental components of a new generation of biosensors that enable the label-free detection of biomarkers [9,10]. Ion-sensitive thin-film transistors (TFTs) have paved the way for novel DNA detection and characterization methods with advantages such as high signal-to-noise ratios, rapid measurement capabilities, and integration into portable instruments [11]. However, they require the creation of a graphene thin film on the biosensor surface through chemical vapor deposition (CVD), which is costly and demanding. CVD also requires high temperatures, along with precise temperature and pressure controls [12]. Organic field-effect transistors (OFETs) have attracted considerable attention in biological detection because of their low cost, flexibility, and light weight nature. In contrast to Si-based FETs, which require high-temperature processing, OFETs can be processed at low temperatures, making them compatible with plastic substrates. Furthermore, techniques such as spin coating, inkjet printing, and screen printing allow for large-area, cost-effective fabrication. The performance characteristics of OFETs can be easily tuned by modifying organic semiconductors, thus offering substantial potential in biosensing technologies. However, organic TFTs are prone to degradation in moisture-laden environments [13,14]. Solution-processed oxide TFTs exhibit strong resilience to moisture degradation, making them an ideal choice for environments with varying humidity levels. Moreover, their simple fabrication through a solution-based method allows for their straightforward and cost-effective production. Thus, aside from their inherent material properties, solution-processed oxide TFTs are a highly economical alternative to their more expensive counterparts, such as GFETs. Moreover, Li et al. reported an interesting solution-based oxide transistor that exhibits transfer conductance two or three orders of magnitude higher [15] than conventional Si-based FETs or GFETs because of the extremely high induced charge density in the channel. They also show a low subthreshold swing factor that approaches the theoretical limit during low-voltage operation. These factors are advantageous for biosensors, showing the potential merits of solution-based oxide transistors.

The detection of DNA or RNA with specific sequences is essential for disease diagnosis and tracking of pathogen subtypes in epidemics. These TFT biosensors enable rapid nucleic acid detection and user-friendly operation compared to PCR, making them valuable tools for efficient and accurate biological detection. Therefore, TFT biosensors may be useful tools for molecular diagnostics and point-of-care applications [8,16].

Recombinase polymerase amplification (RPA) is a type of isothermal amplification technology that can amplify DNA fragments at 37–42 °C for 10–40 min and has less limitation in amplification, which is suitable for point-of-care testing (POCT) of nucleic acid detection in vitro [17,18,19]. In contrast to PCR, RPA does not require a thermal cycle. The entire operation of RPA is very fast, and detectable levels of amplified products can be obtained in less than 40 min.

The heat shock protein 70 gene (*HSP70*) is conserved across prokaryotes and eukaryotes during evolution, making it a suitable target in detecting *Leishmania*, the causative agent of leishmaniasis [20,21,22]. Leishmaniasis is a serious infectious disease that affects the human skin and visceral organs. Its main clinical features include long-term irregular fever, splenomegaly, anemia, emaciation, decreased white blood cell count, and increased serum globulin levels [23,24]. Without proper treatment, certain types of leishmaniasis can lead to death within 1–2 years of infection. Leishmaniasis mainly occurs in Mediterranean countries, as well as in tropical and subtropical regions, with cutaneous leishmaniasis being the most common form. At least 20 *Leishmania* species can be transmitted via the bites of various phlebotomine sandfly species. Although some leishmaniasis types, such as mucocutaneous and visceral leishmaniasis, may cause severe health issues and death, cutaneous leishmaniasis is typically not life threatening and can heal independently in many cases [25,26]. For *Leishmania* detection, LAMP is a widely used isothermal amplification method and has been reported to show similar results to nested PCR [27]. Sereno et al. have reviewed more isothermal amplification methods in parasites of the Trypanosomatidae family, such as NASBA, LAMP, RPA, and PSR, in which RPA and LAMP rank highly in terms of field applicability [28].

In a previous study, we applied a palm-size and one-inch gel electrophoretic device for *Leishmania* analysis of RPA [29]. In the present study, we aimed to develop a rapid and highly sensitive method for detecting *Leishmania* by combining solution-processed oxide TFT biosensors with RPA. The detection ability of the oxide TFT biosensor for RPA was investigated, with the *HSP70* gene as the target DNA for verification.

## 2. Materials and Methods

### 2.1. Appearance and Working Principle of Solution-Processed Oxide TFT

**Figure 1:**
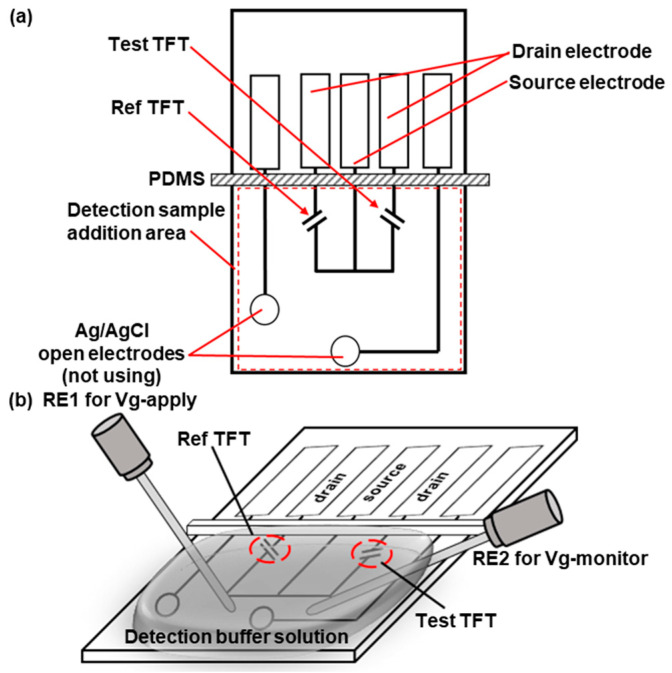
Planar structure of the TFT biosensor (**a**) and setup for Id–Vg curve measurement before and after hybridization (**b**).

Figure 1a shows the planar structure of the TFT biosensor. The TFT biosensor consists of two TFTs. The right-side TFT, referred to as the “Test TFT”, was used for detecting samples. On this TFT, the probe DNA with the complimentary sequence to the target DNA was immobilized to be captured and detected by the TFT. The left-side TFT, referred to as the “Ref TFT”, provided a reference signal when no DNA was specifically bound to it. Therefore, a probe DNA with a sequence that is not complementary to any DNA in the samples was immobilized. Both TFTs shared a common source electrode. Two electrode patterns for Ag/AgCl open reference electrodes were fabricated on the chip for future practical use. In this study, these patterns were not employed, and more accurate reference electrodes are used as mentioned below. Polydimethylsiloxane was used to insulate the electrodes from liquid intrusion. Figure 1b shows the measurement setup for the TFT biosensor. The DNA probes were pre-immobilized on the surface of the Ref and Test TFTs. The source electrode and the two drain electrodes of the TFTs were connected to a 4156C Precision Semiconductor Parameter Analyzer (Keysight, Santa Rosa, CA, USA). A detection buffer solution was added to the “detection sample addition area”. A reference electrode (RE1) was inserted into the solution to apply the gate voltage (Vg-apply). Another standard reference electrode (RE2) was placed in the solution to monitor the actual potential (Vg monitor) applied to the solution. Subsequently, a drain voltage Vd of 200 mV was applied between the source and drain electrodes. Concurrently, a variable voltage Vg ranging from 0 to 800 mV was applied to the gate electrode RE1. The resultant currents flowing through the drains of the Ref and Test TFTs were recorded as Id. The Id–Vg curves were plotted using the Id and Vg-monitor instead of Vg-apply.

Id–Vg curve measurements were performed before and after the DNA sample introduction. If the sample contains the target nucleic acid, it will bind specifically to the DNA probe on the surface of the TFT biosensor.

Nucleic acids are negatively charged particles under the pH conditions of the buffer, and their binding to the probe decreases the current Id value. Therefore, the presence of the target nucleic acid in the sample can be ascertained by comparing the RF and Test TFTs.

**Figure 2:**
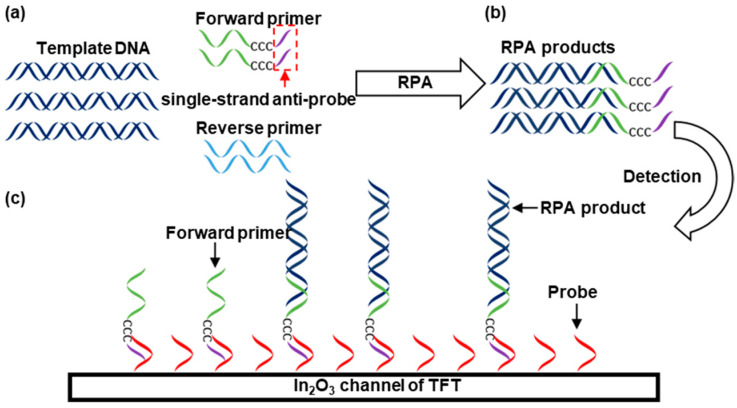
Nucleic acid detection using a TFT biosensor in combination with RPA. From (**a**,**b**), template DNA is amplified by RPA. (**c**) Sample detection.

Figure 2 illustrates the process of nucleic acid detection. A specially designed forward primer was used to enhance the hybridization between the RPA amplicon and probe on the TFT. The forward primer in Figure 2a contains not only the sequence that specifically binds to the template DNA but also a 3-carbon block, namely CCC, and a single-strand anti-probe section. The 3-carbon block was used to prevent the polymerase from extending the DNA chain further, whereas the single-stranded anti-probe remained as a single strand in the amplicon. Therefore, it is expected to bind to the probe on the TFT more effectively than the double-stranded amplicon produced by conventional RPA, PCR, and other methods. The sample with the template DNA was mixed with these primers and RPA reagents, RPA was performed, and the trace target nucleic acid fragments in the sample were amplified in large quantities (Figure 2b). The amplified products carried a single-stranded anti-probe segment from the forward primer. After the RPA, the sample was placed on the TFT biosensor to allow the amplicon to hybridize with the probe on the TFT (Figure 2c). The amplicon can effectively bind to the probe on the TFT because it had a single-stranded binding section. The negative change in the bound amplicon DNA shifted the Id–Vg curve of the TFT in the right-down direction. From this shift, we can detect DNA hybridization. Notably, some unreacted forward primers may have bound to the surface of the TFT biosensor. This effect is discussed in the Results and Discussion section. In this study, a *Leishmania HSP70* fragment was used as the target nucleic acid. The primer sequences are listed in Table 1. [AmC7] stands for [-C_7_H_14_-NH_2_].

### 2.2. Fabrication of Solution-Processed Oxide TFT

**Figure 3:**
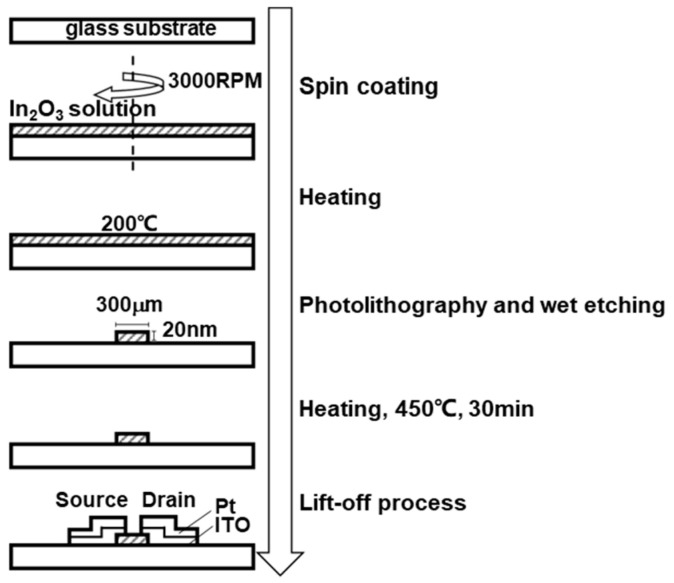
Fabrication of the solution-processed oxide TFT biosensor.

Figure 3 illustrates the TFT fabrication process. Indium(III) Nitrate Trihydrate (FUJIFILM Wako Pure Chemical Corporation, Japan) was dissolved in 2-Methoxyethanol (KANTO CHEMICAL Co., Inc., Japan) to a concentration of 0.2 mol/kg, and acetylacetone (KANTO CHEMICAL Co., Inc., Japan) and ammonium acetate (Sigma-Aldrich, USA) were added to reach a concentration of 0.2 mol/kg. An indium oxide solution was prepared by stirring for 1 h while heating to 100 °C. The indium oxide solution was spin-coated on a glass substrate (Ichikawa Special Glass, Japan) at 3000 rpm and then dried at 200 °C for 20 s on a hot plate. The In_2_O_3_ channel was patterned via photolithography and wet etching using an ITO-02 solution (KANTO CHEMICAL Co., Inc., Japan). The indium oxide film was sintered at 450 °C for 30 min on a hot plate. Subsequently, source and drain electrodes made of indium tin oxide (25 nm) and platinum (80 nm) were fabricated using a sputtering system (ULVAC Co., Ltd., Japan), and a lift-off process was carried out using LOL and TSMR resists (TOKYO OHKA KOGYO Co., Ltd., Japan). After liftoff, the device was washed with EL-grade acetone (KANTO CHEMICAL Co., Inc., Japan) and pure water and then dried with N_2_ blow. After annealing, the substrate was baked at 400 °C for 30 min on a hot plate.

### 2.3. TFT Biosensor Modification

The initial stage involved cleaning the TFT biosensor with deionized water to remove contaminants. Then, a nitrogen gun was employed to thoroughly dry the TFT biosensor for later use.

The biosensor was subjected to plasma ashing at 30 W for 15 s to remove organic residues and surface contaminants. It was then treated with 1% (3-glycidox-ypropyl)trimethoxysilane (GPTMS) at room temperature for 30 min. To conclude this phase, the TFT biosensor was rinsed with deionized water to remove excess GPTMS and then thoroughly dried with a nitrogen gun.

The second stage involved the fixation of the DNA probe. A Petri dish was prepared and layered with deionized water to ensure a moist environment. The TFT biosensor was spread on a moist paper towel, and 1 µL of the probe solution was added dropwise onto the semiconductor area of the chip. This step was performed at room temperature, and the chip was subsequently fixed for 30 min.

In the third and final stage, the TFT biosensor was thoroughly rinsed with deionized water to remove the probe solution and then dried with a nitrogen gun. Subsequently, ethanolamine was added to the chip surface and left for 30 min. To finalize the process, the TFT biosensor was rinsed with ultrapure water and dried with a nitrogen gun.

### 2.4. RPA Sample Preparation

The *Leishmania HSP70* template was synthetically prepared from the parasite of the *Leishmania* major species using a conventional PCR method (Jichi Medical University). The concentration of the *Leishmania HSP70* template was determined using Nanodrop2000 (Thermo Fisher Scientific, USA). The template was then diluted with a dilution buffer (EASY Dilution, Takara Bio Inc., Japan), yielding sample concentrations ranging from 10^6^ to 10^1^ copies/µL. The subsequent step involved the configuration of the RPA reaction system. The TwistAmp^®^ Liquid Basic (RPA kit) was purchased from TwistDxTM (Scarborough, ME, USA). The measurement buffer consisted of 1 mM phosphate buffer (PB buffer) with 30 mM NaCl. In addition, 1× PBS was diluted from 10× PBS (Nippon, Japan).

Mixing of the provided reagents in the RPA kit, which included a buffer, a polymerase, and a recombinase, was carried out. Then, we added dNTP and designed primers. To this system, 1 µL of the template at different concentrations of 10^6^–10^1^ copies/µL was added. The next stage involved an interaction between magnesium acetate and the RPA reaction system. After the magnesium acetate was sufficiently mixed into the system, the centrifuge tubes containing the reaction mixture were swiftly transferred to a 42 °C incubator. The samples were then incubated for 20 min, during which the RPA reaction occurred.

Following the incubation, an ice pack was used to rapidly cool down the centrifuge tubes and halt the RPA reaction. Subsequently, proteinase K was added to all the tubes to effectively break down the RPA amplification enzyme and terminate the RPA reaction completely. The tubes were shaken, centrifuged for a few seconds, and then incubated at 37 °C for 10 min. This step allowed proteinase K to degrade the SSB-DNA complex, thereby completing the process.

### 2.5. Evaluation of the Effects of Proteinase K Treatment on Experimental Outcomes

In POCT, rapid detection and accuracy are crucial for detecting the presence of a target gene in a sample. In the present study, three types of RPA samples were prepared: untreated, proteinase K-treated, and purified RPA products. For the first type, we hypothesized that the accurate detection of untreated RPA products by the TFT biosensor would significantly streamline the detection process, reduce the detection time, and minimize reagent consumption. For the second type, we hypothesized that treatment with proteinase K would increase the detection accuracy and sensitivity, considering that this enzyme is a broad-spectrum serine protease often used to digest proteins and remove potential inhibitors. For the third type, we hypothesized that the purification of RPA-amplified products by using a PCR purification kit can improve the accuracy of the results by eliminating potential inhibitors, impurities, and unreacted primers.

### 2.6. Detection of Leishmania Using the TFT Biosensor

*Leishmania* was detected using the prepared TFT biosensor and RPA products. The RPA products were incubated for amplification separately in tubes, then placed on TFT biosensors for incubation for hybridization. For each trial, the Id–Vg curve was measured three times before incubation with the RPA product for hybridization. After incubation, the Id–Vg curves were measured thrice. The stability and shift in the curves were then evaluated.

For one sample measurement, we rinsed the surface of the chip with a measurement buffer solution (1 mM PB with 30 mM NaCl), dried the chip surface with a dust removal gun, and then connected the electrodes to the TFT biochip. The entire detection area was covered with measurement buffer solution. The Id-Vg curve was recorded in the Vg range of 0–0.8 V.

The chip was washed with deionized water and then dried using a dust removal gun. On a Petri dish, the TFT biosensor was positioned on a layer of wet paper. A 1 mL aliquot of the RPA-amplified sample was added to the two semiconductor channels, and the Petri dish was covered to prevent rapid drying. The dish was incubated at room temperature for approximately 15 min to hybridize the target DNA and probes. The TFT biosensor was transferred to a new Petri dish and then added to 1 × PBS. The Petri dish was gently shaken to remove excess samples. Then, the TFT biosensor was rinsed with the 1 mL measurement buffer 3 times and dried with a dust removal gun. Similarly, the electrodes of the parameter device were connected to the TFT biosensor. The measurement buffer was added, and detection was performed a second time to obtain the voltage–current change graph after DNA sample hybridization.

## 3. Results and Discussion

### 3.1. Structure and Working Principle of Solution-Processed Oxide TFT

**Figure 4:**
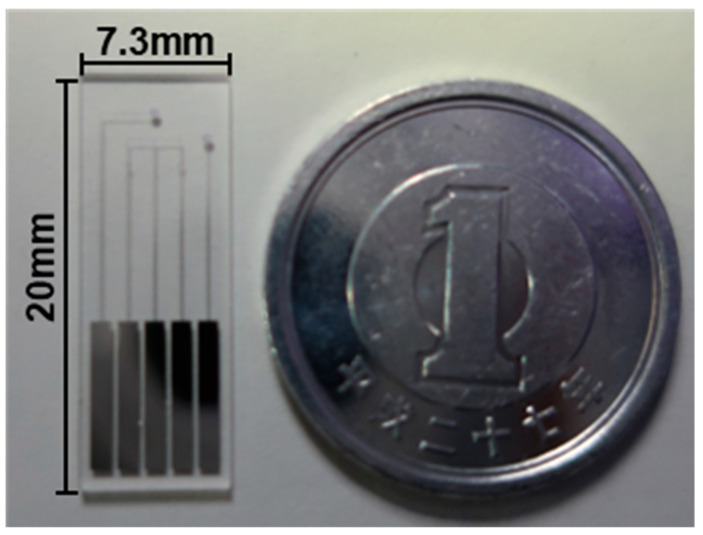
Photo of the fabricated TFT biosensor.

Figure 4 shows the photo of the fabricated TFT biosensor, measuring 20 mm in length and 7.3 mm in width, slightly smaller than the size of a 1-yen coin.

### 3.2. RPA Reaction System Optimization on Experimental Outcomes

**Figure 5:**
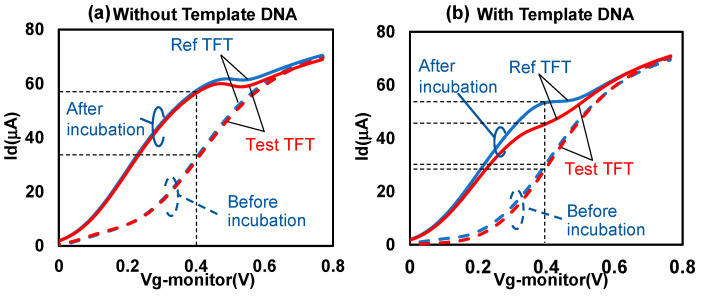
Id variation in (**a**) purification RPA products without template DNA and (**b**) purification RPA products with template DNA.

Figure 5 depicts the typical changes in the Id–Vg curve of the TFT biosensor before and after incubation with the RPA products. The products were treated with proteinase K and purified before incubation. Measurements were performed three times before and after incubation. Here, the curves represent the third measurement. In Figure 5a, the RPA reaction system did not contain template DNA (replaced with the same amount of ultrapure water), whereas in Figure 5b, the RPA reaction system contained 10^3^ copies/μL template DNA. Figure 5a,b show that the Id value increased after incubation. However, as shown in Figure 5a, no significant difference in the Id values of the Ref and Test TFTs were found before and after incubation. By contrast, in Figure 5b, after incubation with the purified RPA samples, the Id value of the Test TFT significantly decreased compared with that of Ref TFT, especially at Vg values near 0.4 V.

**Figure 6:**
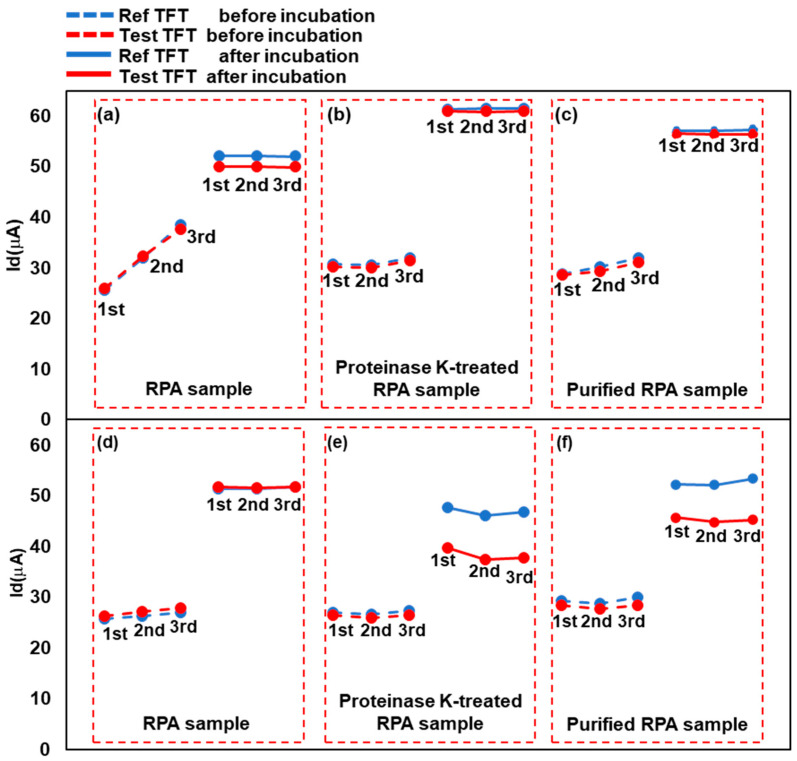
Change in current before and after sample addition. The three values on the left are pre-incubation readings, whereas those on the right are post-incubation readings. The detected RPA samples were (**a**,**d**) untreated RPA-amplified products, (**b**,**e**) proteinase K-treated RPA products, and (**c**,**f**) purified RPA products. In each graph, “Ref TFT” denotes the reference TFT, which will not bind with the target DNA in the sample, whereas “Test TFT” refers to the Test TFT, capable of binding with the target DNA segment. In (**a**–**c**), ultrapure water instead of template DNA was added to the RPA reaction solutions. In (**d**–**f**), template DNA was added to all RPA solutions.

Figure 6 summarizes the change in Id at Vg = 0.4 V under the various conditions listed in Table 2. Measurements were performed three times before and after incubation under all conditions. As shown in Figure 6a–c, regardless of the type of RPA sample, no significant change in Id was found between the Ref and Test TFTs. As shown in Figure 6e–f, the Id value in the Test TFT noticeably decreased in the proteinase K-treated and purified RPA samples but did not decrease in the untreated RPA product. The magnitudes of the changes shown in Figure 6e,f were approximately equivalent. We considered that this is because of the effect of single-stranded binding proteins (SSB) in the RPA reagents. In RPA, the SSB protein binds to single-stranded DNA during the amplification process [30,31], protecting it from degradation and preventing the formation of unwanted secondary structures, thus promoting DNA amplification. The SSB protein interacts with single-stranded DNA to form a more stable protein–nucleic acid complex than DNA–DNA, protecting the single-stranded DNA and increasing amplification efficiency and accuracy. However, because of the presence of SSB, the amplified DNA may not bind well to the probes on the chip surface. Proteinase K is expected to degrade SSB proteins in the RPA samples. These results indicate that the TFT biosensor preferentially detected the RPA product, whereas the TFT responded less to the forward primer. This finding may be due to the differences in DNA length. Here, the Id values before incubation were expected to be the same because they were taken in the same condition. In fact, in Figure 6, they changed slightly mainly due to the deviation during the device fabrication process, but were almost close and this indicates a level of reproducibility of our TFT devices. For reproducibility, it is important to note the Id differences in pair of Ref TFT and Test TFT on a biosensor device. They were a few µA level and small enough differences for the above discussions.

### 3.3. Leishmania HSP70 Detection by the TFT Biosensor

**Figure 7:**
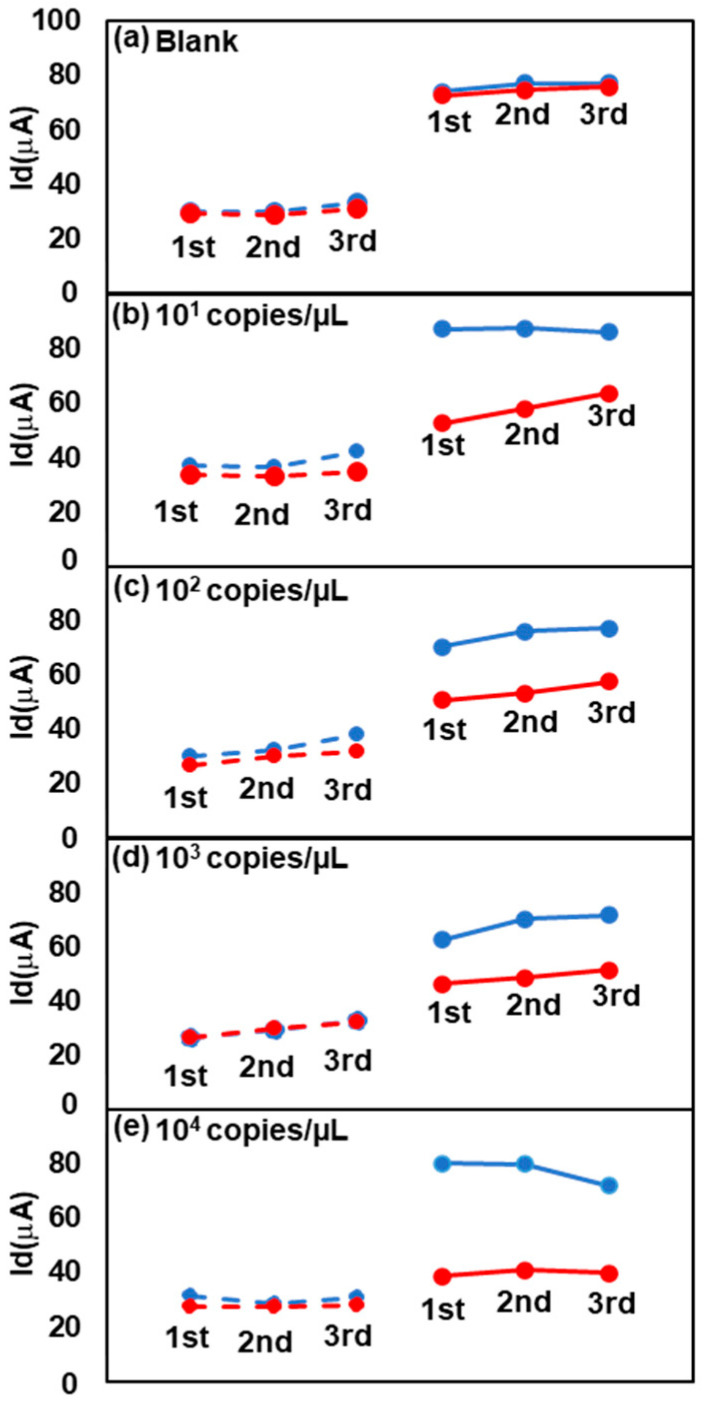
Change in current values for the positive and negative groups at the 0.4 V position before and after incubation. The template DNA concentrations are (**a**) 0 copies/μL, (**b**) 10^1^ copies/μL, (**c**) 10^2^ copies/μL, (**d**) 10^3^ copies/μL and (**e**) 10^4^ copies/μL. RPA conditions at 42 °C for 20 min, with the addition of 1% SDS after the amplified samples were treated with proteinase K.

Figure 7 illustrates the results obtained from assessing the TFT biosensor using RPA-amplified products at different concentrations of 10^1^–10^4^ copies/μL, complemented by a blank control group, under identical RPA reaction conditions (42 °C for 20 min). The primary distinction lies in the application of proteinase K. Following the RPA reaction, 3 μL of proteinase K and 3 μL of 1% sodium dodecyl sulfate (SDS) were incorporated into the reaction system and subsequently incubated at 37 °C for 10 min. The results revealed a more pronounced decrease in the current of the positive sample after SDS treatment than that without SDS treatment (Figure S1). This was also considered to be caused by the SSB. SDS may denature the SSB more completely together with proteinase K and improve the binding between the RPA amplicon and the probe DNA on the TFT. Thus, the combination of the RPA and TFT biosensors was able to detect *L. braziliensis HSP70* at a concentration of at least 10^1^ copies/μL, demonstrating highly sensitive detection within 20 min of isothermal amplification.

In this study, we used RPA amplification products containing specific nucleotide sequences for DNA detection. The primers were designed using the 5′-TACACAGCAC[CCC]-3′ sequence, where [CCC] is a three-carbon structure that can block DNA replication by extending beyond the primer’s extra sequence. Therefore, the amplified product contains a 5′-TACACAGCAC-3′ fragment of the amplified nucleic acid, which can specifically bind to the surface of the chip containing the 5′-GTGCTGTGTA-3′ sequence. Thus, the *HSP70* gene fragment was converted into 5′-TACACAGCAC-3′ and 5′-GTGCTGTGTA-3′ sequences. This approach not only eliminates the need for probe design but also increases the versatility of TFT chips for nucleic acid detection, which can be applied to the detection of other nucleic acid fragments.

All Id values increased after incubation. This result is speculated to be due to the nonspecific binding of some substances in the RPA reagents. This increase can be canceled out by comparing the Id values of the Ref and Test TFTs.

The amplification efficiency was low when the template DNA was low, which resulted in less primer consumption. Thus, in the low- and zero-concentration cases, most of the bound molecules on the chip surface were forward primers rather than RPA products. However, a greater decrease in Id was observed at higher template concentrations (Figure 7). This result indicates that the sensitivity of TFT for primers was much lower than that for RPA amplicons. The primer length was short, approximately 20 bases, in comparison with the *HSP70* nucleic acid fragments, which were as long as 280 bp.

As shown in Figure S1c, the sensor responds to the concentration range from 10 to 10^4^ copies/µL. From 10^4^ to 10^6^ copies/µL, the response is nearly saturated but distinguishable from the concentration below 10^4^ copies/µL. Figure S3 shows the measurement results for samples including the *Escherichia coli (E. coli)* 16S rRNA gene by the developed method for *Leishmania*. The Id values from both the Test TFT and Ref TFT for two different concentrations of the 16S rRNA gene were almost identical. These results mean that the TFT biosensors did not respond to the *E. coli* 16S rRNA gene, as the TFT biosensors specifically responds to the *HSP70* gene of *Leishmania*.

## 4. Conclusions

We successfully designed and fabricated a TFT biosensor using inorganic materials and glass substrates. The prepared TFT biosensor can realize rapid nucleic acid detection by monitoring current changes before and after sample hybridization. This innovative approach provides a novel method for the rapid diagnosis of infectious diseases. Furthermore, the use of inorganic materials and glass substrates allows the rapid and cost-effective production of this biosensor via printed circuits. It also enables a longer lifetime and ease of operation, owing to its moisture tolerance compared to organic TFT. Notably, the sequence of the probe on TFT is independent from the target DNA. It means that only changing the primers, one of whom has single-strand anti-probe, is requiredfor new targets. The TFT chip with a constant probe demonstrates a wide range of applicability and can be commonly produced in advance. This feature enhances our research efficiency and design investment. Furthermore, the TFT biosensors do not require larger instruments such as fluorescence microscope to observe the results.

By combining the TFT biosensor with the isothermal amplification technology, RPA, we were able to detect the *Leishmania HSP70* fragment, achieving an impressive detection of 10^1^ copies/μL. Moreover, it is not affected by non-target amplification products. Compared with traditional gel electrophoresis and fluorescence detection, this TFT biosensor can significantly improve the detection capability of target nucleic acids.

However, despite the significant progress that we have made, our work is far from complete. Although our TFT biosensor can detect trace levels of nucleic acids in combination with RPA, its current performance still requires further optimization. This area requires continuous effort and exploration. Therefore, we will seek new methods and strategies to further enhance the stability and performance of the TFT biosensor.

## Figures and Tables

**Table 1:** Sequence information of the *Leishmania HSP70* gene, primers, and probe immobilized on the biosensor surface.

Name	Sequence
*Leishmania* Heat shock protein 70 gene	5′−CATATCACCATCACCAACGACAAGGGCCGACTGAGCAAGGACGAGATCGAGCGCATGGTGAACGATGCGTCGAAGTACGAGCAGGCCGACAAGATGCAGCGCGAGCGCGTGGAGGCGAAGAACGGCCTGGAGAACTACGCGTACTCGATGAAGAACACGGTCTCCGACACGAACGTGTCCGGCAAGCTGGAGGAGAGCGACAGGTCCGCGCTGAACTCGGCGATCGACGCGGCGCTGGAGTGGCTGAACAGCAACCAGGAGGCGTCGAAGGAAGAGTACGAGCA−3′
Forward primer	LB-hsp70-sp-Fwd 5′−TACACAGCAC[CCC]CATATCACCATCACCAACG−3′
Reverse primer	LB-hsp70-sp-Rev 5′−TGCTCGTACTCTTCCTTCG−3′
Probe	Anti-capture-NH_2_ 5′−GTGCTGTGTATTTTTT−[AmC7]−3′

**Table 2:** RPA sample processing conditions.

	a	b	c	d	e	f
Template DNA	-	-	-	+	+	+
Proteinase K	-	+	-	-	+	-
Purification	-	-	+	-	-	+

## Data Availability

Data are contained within the article.

## Supplementary Materials

The following supporting information can be downloaded at: https://www.mdpi.com/article/10.3390/bios13080765/s1, Figure S1. Change in Id before and after incubation for the positive (Test TFT) and negative (Ref TFT) groups at the 0.4 V position. These values are the averages of Id-Vg measurements (N = 3) taken three times. Error bars represent the standard deviation. (a) was initially amplified by RPA at 42 °C for 10 min, while (b) and (c) were initially amplified by RPA at 42 °C for 20 min. After RPA, the samples were treated with the addition of proteinase K (a), and with the addition of proteinase K and 1% SDS (b) and (c), at 37 °C for 10 min. Figure S2. The 6% polyacrylamide gel electrophoresis of RPA products at initial template DNA concentrations of 10^10^–10^7^ copies/µL. Figure S3. Change in Id before and after incubation for the positive (Test TFT) and negative (Ref TFT) groups at the 0.4 V position. These values are the averages from Id-Vg measurements (N = 3) taken three times. Error bars represent the standard deviation. The templates of *E. coli* concentrations are 0 μM, 1 μM and 10 μM, respectively. They were initially amplified by RPA at 42 °C for 20 min, and then the samples were treated with the addition of proteinase K and 1% SDS at 37 °C for 10 min.
