# Supplementary material for: Rapid and Highly Sensitive Detection of Leishmania by Combining Recombinase Polymerase Amplification and Solution-Processed Oxide Thin-Film Transistor Technology
Source: Biosensors (Basel). 2023 Jul 28;13(8):765. doi: 10.3390/bios13080765 (PMC10452724; doi:10.3390/bios13080765)
Supplement: Supplementary file 1 [file biosensors-13-00765-s001.zip › biosensors-2475819-supplementary.pdf]

Supporting Information

Rapid and Highly Sensitive Detection of *Leishmania* by Combining Recombinase Polymerase Amplification and Solution-Processed Oxide Thin-Film Transistor Technology

Weidong Wu [1], Manish Biyani [1,2], Daisuke Hirose [1] and Yuzuru Takamura [1,*]

[1] School of Materials Science, Japan Advanced Institute of Science and Technology, Nomi 923-1292, Ishikawa, Japan; weidong.wu@ml.jaist.ac.jp (W.W.); biyani@jaist.ac.jp (M.B.); d-hirose@jaist.ac.jp (D.H.)
[2] BioSeeds Corporation, JAIST Venture, Ishikawa Create Laboratory 202, Asahidai 2-13, Nomi 923-1292, Ishikawa, Japan
[*] Correspondence: takamura@jaist.ac.jp

Figure S1 presents the Id differences between before and after incubation with RPA samples at different template concentration of 0 and 10^1–10^3 copies/μL. These experiments were conducted under identical RPA reaction conditions, (a)42°C for 10 min, (b) and (c) 42°C for 20 min. After the RPA reaction, the reaction system was supplemented with 3 μL of proteinase K for (a) and (b), and with 3 μL of proteinase K and 3 μL of 1% SDS for (c), followed by incubation at 37°C for 10 min. Figure S1(a) shows that the RPA reaction time of 10 min is not enough for detection of 100 copies/μL. In Figure S1(b) with 20 min of the RPA reaction time, we could observe significant differences in Id values for all the analyzed template DNA concentrations of 10 to 10^4 copies/μL. We avoided further longer incubation time as we have observed that non-specific amplification (by-products mainly by primer-dimer reactions) in RPA always increases with increasing the time of incubation above 30 minutes [Anal Methods 2019: 11, 29, 4953-5072; Sci. Rep. 2021: 11, 15997]. Therefore, we decided to choose 20 min incubation time in this study. Figures S1(b) and (c) show that more significant reduction in Id for samples treated with SDS compared to the group without SDS.

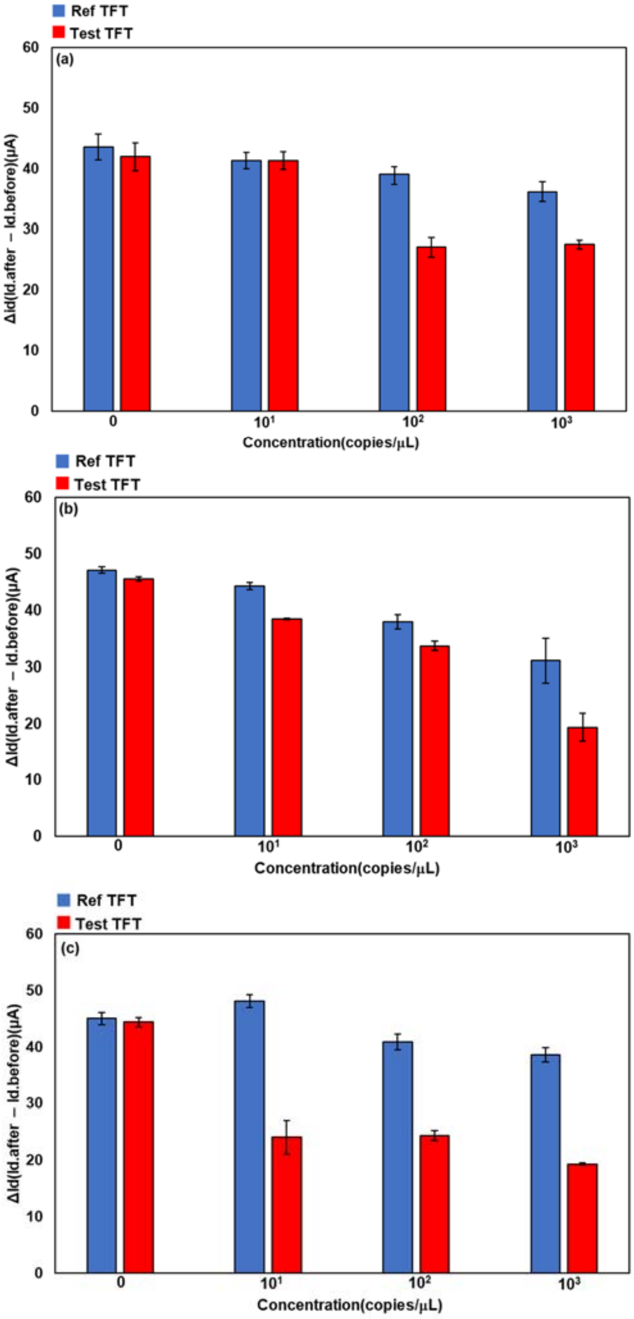

Figure S1. Change in Id before and after incubation for the positive (Test TFT) and negative (Ref TFT) groups at the 0.4 V position. These values are the averages from 3 times Id-Vg measurement (N=3). Error bars represent the standard deviation. (a) was initially amplified by RPA at 42℃ for 10 min, while (b) and (c) was initially amplified by RPA at 42℃ for 20 min. After RPA, the samples were treated with the addition of proteinase K (a)(b), and with the addition of proteinase K and 1% SDS (c), at 37°C for 10 min.

Figure S2 shows the results of 6% polyacrylamide gel electrophoresis of the RPA product at initial template DNA concentrations of 10^{10}–10^{7} copies/μL. At template DNA concentrations below 10^{9} copies/μL, the amplified products could not be detected using gel electrophoresis.

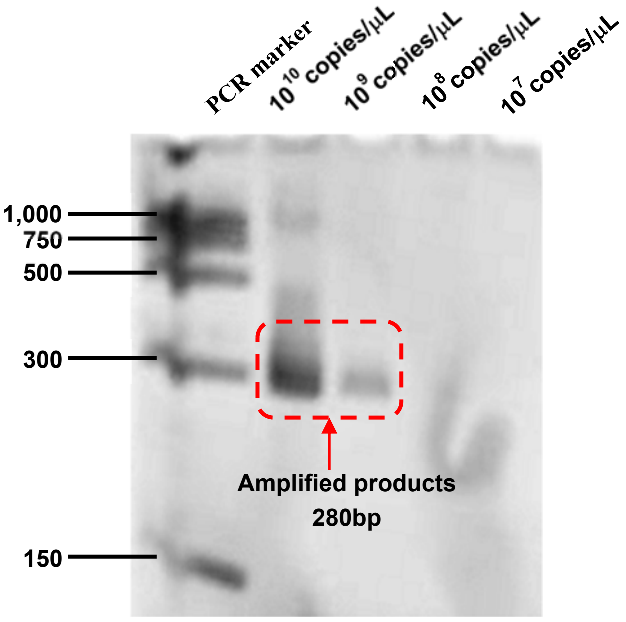

Figure S2. 6% polyacrylamide gel electrophoresis of RPA products at initial template DNA concentrations of 10^{10}–10^{7} copies/μL.

Figure S3 shows the measurement results for samples including *E.coli* 16S rRNA gene by the developed method for *Leishmania*. The Id values from both the Test TFT and Ref TFT for two different concentrations of 16S rRNA gene were almost identical. These results confirm that the TFT biosensors didn't show non-specific signals.

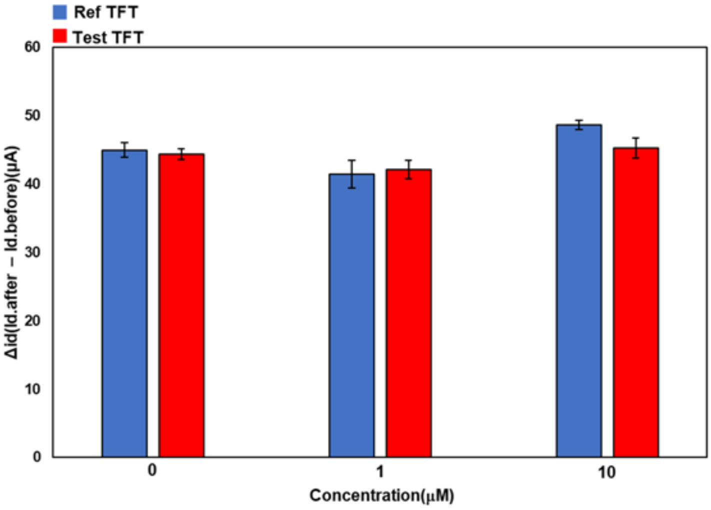

Figure S3. Change in Id before and after incubation for the positive (Test TFT) and negative (Ref TFT) groups at the 0.4 V position. These values are the averages from 3 times Id-Vg measurement (N=3). Error bars represent the standard deviation. The template of *E.coli* concentration are 0μM, 1μM and 10μM, respectively. They was initially amplified by RPA at 42℃ for 20 min, then the samples were treated with the addition of proteinase K and 1% SDS at 37°C for 10 min.
